# Risk Factors, Trends, and Preventive Measures for 30-Day Unplanned Diabetic Ketoacidosis Readmissions in the Pediatric Population

**DOI:** 10.7759/cureus.19205

**Published:** 2021-11-02

**Authors:** Deepa Vasireddy, Mukul Sehgal, Amod Amritphale

**Affiliations:** 1 Pediatrics, Pediatric Group of Acadiana, Lafayette, USA; 2 Critical Care Medicine, University of South Alabama, Mobile, USA; 3 Medicine/Cardiovascular Disease, University of South Alabama College of Medicine, Mobile, USA

**Keywords:** type 2 diabetes, pediatric critical care, type 1 diabetes mellitus, database hcup nis nrd research, insulin, thyroid disorder, depression in chronic illness, 30 day readmissions, diabetic ketoacidosis (dka)

## Abstract

Background

There has been a steady rise in types 1 and 2 diabetes mellitus among the youth in the USA from 2001 to 2017. Diabetic ketoacidosis (DKA) is a common and preventable presentation of both types of diabetes mellitus. According to the Centers for Disease Control and Prevention’s (CDC) United States Diabetes Surveillance System, during 2004-2019 an increase in DKA hospitalization rates by 59.4% was noted, with people aged less than 45 years having the highest rates. Readmissions reflect the quality of disease management, which is integrally tied to care coordination and communication with the patient and their families. This study analyzes the trends and risk factors contributing to 30-day unplanned DKA readmissions in the pediatric age group and looks into possible preventive measures to decrease them.

Methods

A retrospective study was performed using the National Readmission Database (NRD) from January 1, 2017, to December 1, 2017. Pediatric patients aged 18 years and younger with the primary diagnosis of DKA were included using the International Classification of Diseases, Tenth Revision, Clinical Modification (ICD-10-CM) code E10.10. All statistical analysis was performed using IBM SPSS Statistics for Windows, version 1.0.0.1327 (IBM Corp., Armonk, NY, USA). Pearson’s chi-square test was used for categorical variables and Mann-Whitney U test was used for continuous variables. To independently determine the predictors of readmission within each clinical variable, multiple logistic regressions with values presented as odds ratios (OR) with 95% confidence intervals (CI) were performed.

Results

A weighted total of 19,519 DKA-related pediatric index admissions were identified from the 2017 NRD. Of these pediatric patients, 831 (4.3%) had 30-day DKA readmission. The median age of a child for readmission was 16 years with an interquartile range of 0 to 18 years. A sharp rise in 30-day DKA readmissions was noted for ages 16 years and over. Females in the 0-25th percentile median household income category, with Medicaid covered, large metropolitan areas with at least 1 million residents, and metropolitan teaching hospitals were found to have a statistically significant higher percentage of readmissions. The mean length of stay for those who had a DKA readmission was 2.06 days, with a standard deviation of 1.84 days. The mean hospital charges for those who had a DKA readmission were $ 20,339.70. The 30-day DKA readmission odds were seen to be increased for female patients, Medicaid-insured patients, admissions at metropolitan non-teaching hospitals, and children from 0-25th percentile median household income category.

Conclusion

There has not been much of a change in the trend and risk factors contributing to the 30-day unplanned DKA readmissions over the years despite the steady rise in cases of diabetes mellitus. The length of stay for those who did not get readmitted within 30 days was longer than for those who did. This could reflect more comprehensive care and discharge planning that may have prevented them from readmission. Diabetes mellitus is a chronic disease that demands a team effort from the patient, family, healthcare personnel, insurance companies, and lawmakers. There is scope for a lot of improvement with the way our patients are being managed, and a more holistic approach needs to be devised.

## Introduction

There has been a steady rise in type 1 diabetes mellitus (T1DM) and type 2 diabetes mellitus (T2DM) among the youth in the USA. Type 1 DM is the most common type of DM in the youth in the USA [1]. From 2001-2017, there has been an increase in children aged 5-19 years with T1DM across both sexes and is more common in white youth, though an overall increase has been noted across each racial and ethnic group. T2DM has also shown an increase in cases in those in the age group of 10-19 years in both sexes and is more common in racial and ethnic minority groups, though an overall increase has been noted across each racial and ethnic group as well [2]. Diabetic ketoacidosis (DKA) is a common preventable presentation of both types of diabetes mellitus.

Those with established T1DM are at a risk of DKA when they miss their insulin doses due to either poor compliance or pump failure. Sepsis, gastrointestinal illnesses causing diarrhea or vomiting, and trauma due to a rise in counter-regulatory hormone can increase a diabetic patient’s risk of DKA. Poor dietary compliance or eating disorders and consumption of high carbohydrate content intake are other risk factors. Children with prior poor diabetic control or prior episodes of DKA poor family support and with poor access to medical care services are placed at an increased risk of DKA [3]. According to one study, genetic and immunological markers seemed to have an association with risk of DKA in T1DM [4]. Certain antipsychotic medications such as clozapine, olanzapine, and risperidone can cause DKA. Alcohol and cocaine use have been linked to DKA, as well as corticosteroids and some other medications [5].

According to the Centers for Disease Control and Prevention’s (CDC) United States Diabetes Surveillance System, during 2004-2019 an increase in DKA hospitalization rates by 59.4% was noted, with people aged less than 45 years having the highest rates [6]. Mortality in DKA is predominantly associated with the development of cerebral edema as a complication, occurring in about 0.3-1% of all cases [7]. DKA leads to long hospital stays and high healthcare costs. According to a recent study on the National Inpatient Sample (NIS) data, a higher incidence of DKA was found in the age group of 1-17 years per 10,000 admissions. Across age groups, males had a higher mortality. Blacks had the higher number of DKA cases and deaths per 10,000 admissions and 10,000 cases, respectively. A further rise was noted in the incidence of DKA per 10,000 admissions from 2014 to 2017 [8]. According to another study, mean hospital charges after adjusting for inflation per admission had shown an increase from 2003 to 2014; the rise was from $18,987 to $26,566 from 2003 to 2014. The average length of stay (LOS) had shown a slight decline over these years [9]. The mean hospital charges had shown a further rise into 2017 and the LOS showed a slight decrease since. Across age groups, Medicaid patients had the highest DKA cases per 10,000 admissions and Medicare patients had the higher deaths per 10,000 DKA patients admitted [8].

Looking specifically into DKA readmissions, in one study it was noted that lower socioeconomic status (SES) and Medicaid coverage had a stronger association with DKA readmissions in the pediatric age group. In the case of readmissions, females were found to have a higher odds ratio in the pediatric age group [10]. In a Nationwide Readmission Database (NRD) study in 2018, T1DM patients with DKA were found to have higher 30-day readmission rates [11]. Particularly in children, there is a wide variance in resource use, LOS, and readmission rates in patients with DKA across U.S. children’s hospitals [12]. Readmissions reflect the quality of disease management, which is integrally tied to care coordination and communication with the patient and their families. Given the healthcare burden of DKA, we wanted to specifically analyze trends and risk factors contributing to 30-day unplanned DKA readmissions that contribute to the current situation in the pediatric age group and look into possible preventive measures to curb them.

## Materials and methods

Study design

A retrospective study was performed using an all-patient publicly available database for the year 2017. Diagnosis codes pertaining to DKA were used to identify index admissions. Our study was exempted from the Institutional Review Board (IRB) at the University of South Alabama as we used a publicly available database. We followed the well-described methodology in prior studies [13-16].

Data source

We obtained data using the National Readmission Database (NRD) from January 1, 2017, to December 1, 2017. It is the largest all-patient, all-payer inpatient database available in the United States and is developed and maintained by the Agency for Healthcare Research and Quality for the Healthcare Cost and Utilization Project (HCUP). It includes publicly available hospitalization data from non-federal hospitals. The 2017 NRD includes data from 28 states and approximately 5 million pediatric discharges. Overall, 58.2% of all hospitalizations in the year 2017 and 60% of the population are represented in the database. Readmission within a state and a given year and tracked using unique patient identifiers that are allotted to each admission. Patient and hospital demographics, diagnostic and procedural codes assigned at the time of discharge are available in the NRD.

Population

We identified pediatric patients aged 18 years or below who had a primary diagnosis of DKA using the International Classification of Diseases, Tenth Revision, Clinical Modification (ICD-10-CM) codes. We utilized code E10.10. Patients who did not survive the index admission or had index discharge in the month of December were excluded from the study.

Covariates

Patient demographics chosen for the index admission included age, gender, insurance coverage, median household income, All Patients Refined Diagnosis Related Groups (APRDRG) severity of illness, and discharge disposition. Comorbidities were also included using ICD-10-CM codes. Hospital demographics included designation (large, small, micropolitan, non-urban) and teaching status.

Statistical analysis

All statistical analysis was performed using IBM SPSS Statistics for Windows, version 1.0.0.1327 (IBM Corp., Armonk, NY, USA). Variables such as age, gender, household income, insurance coverage, and loss of function were tested for statistical differences using Pearson’s chi-square test for categorical variables and Mann-Whitney U test for continuous variables, with the reference group as those who did not have a readmission. Clinical predictors for 30-day readmission were analyzed using multivariable logistic regression. To independently determine the predictors of readmission within each clinical variable, multiple logistic regressions with values presented as odds ratios (OR) with 95% confidence intervals (CI) were performed.

## Results

A weighted total of 19,519 DKA-related pediatric index admissions were identified from the 2017 NRD. Of these pediatric patients, 831 (4.3%) had 30-day readmission. Of them, 99.8% of the readmissions were T1DM patients and 0.2% were T2DM patients. Sociodemographic and socioeconomic factors of the pediatric population were studied (Table 1).

**Table 1:** Socioeconomic and sociodemographic characteristics of pediatric patients with 30-day DKA readmissions Frequencies of variables were rounded to the closest whole number, and missing data numbers were excluded. APRDRG, All Patients Refined Diagnosis Related Groups); DKA, diabetic ketoacidosis

Variables	30-day DKA readmissions	
	Frequency of variable (% of total frequency)	
	Yes	No
Gender		
Male	347 (41.76)	9350 (50.03)
Female	484 (58.23)	9338 (49.96)
Median household income category		
0-25th percentile	375 (45.09)	5801 (31.04)
26-50th percentile	209 (25.12)	5493 (29.39)
51-75th percentile	152 (18.22)	4541 (24.29)
76-100th percentile	86 (10.36)	2659 (14.23)
Type of insurance coverage		
Medicare	2 (0.24)	27 (0.14)
Medicaid	569 (68.37)	9983 (53.41)
Private	200 (24.07)	7518 (40.23)
Self-pay	18 (2.15)	446 (2.38)
No charge	40 (4.85)	8 (0.04)
Other	0	685 (3.67)
APRDRG severity of illness		
No class specified	None	2 (0.01)
Minor loss of function (includes cases with no comorbidity or complications)	None	78 (0.42)
Moderate loss of function	678 (81.53)	15826 (84.68)
Major loss of function	134 (16.11)	2523 (13.49)
Extreme loss of function	20 (2.36)	258 (1.38)
Hospital urban-rural categories		
Large metropolitan areas with at least 1 million residents	447 (53.70)	10338 (55.31)
Small metropolitan areas with less than 1 million residents	333 (40.10)	7539 (40.34)
Micropolitan areas	37 (4.59)	628 (3.36)
Not metropolitan or micropolitan (non-urban residual)	14 (1.71)	183 (0.98)
Hospital location and teaching status		
Metropolitan non-teaching	105 (12.65)	1489 (7.96)
Metropolitan teaching	675 (81.17)	16388 (87.70)
Non-metropolitan non-teaching	51 (6.17)	811 (4.33)

**Figure 1:**
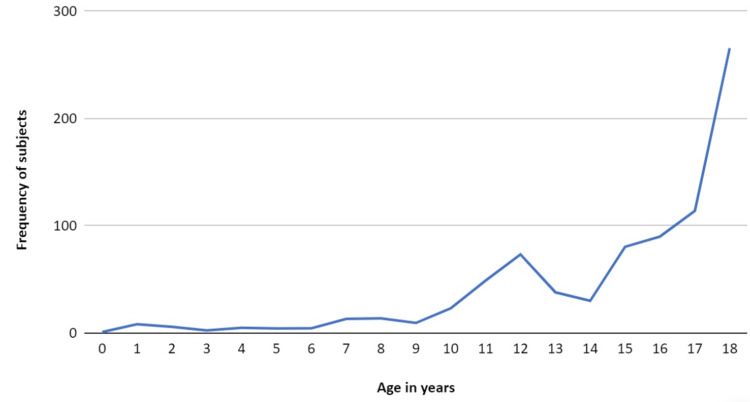
Age-based trend of diabetes ketoacidosis readmissions

Infancy to age 18 years was the age range considered for this study. The median age of a child for readmission was 16 years, with an interquartile range of 0 to 18 years. The trend of DKA readmissions according to the age of the child is shown in Figure 1.

A sharp rise in 30-day DKA readmissions was noted for ages 16 years and over. The age group of 12-18 years contributed to 83.1% of the total readmissions; 58.2% of the readmissions were female. The median household income the patient resided in was considered for the purposes of this study, and 45.1% of the DKA readmissions were from the 0-25th percentile category. Patients with Medicaid insurance formed the greatest percentage (68.4%) of the readmissions among the various insurance plans the patients held. Based on the APRDRG severity of illness, 81.5% of the DKA readmissions had moderate loss of function.

Patients from large metropolitan areas with at least 1 million residents formed the greatest percentage (53.7%) of DKA readmissions when considering the designation of the hospital the admission occurred at. Metropolitan teaching hospitals contributed to 81.2% of the DKA readmissions. The mean LOS for those who had a DKA readmission was 2.06 days, with a standard deviation of 1.84 days. The mean LOS for those who did not have a readmission was 2.28 days, with a standard deviation of 2.77 days. The mean hospital charge for those who had a DKA readmission were $20,339.70 and that for those who did not have a readmission were $ 22,189.50.

Logistic regression was implemented, and 30-day readmission odds were seen to be increased for female patients, Medicaid-insured patients, admissions at metropolitan non-teaching hospitals, and children from the 0-25th percentile median household income category (Table 2).

**Table 2:** Multivariable logistic regression analysis with OR and 95% CIs for variables associated with 30-day diabetes ketoacidosis readmissions -^a^: insufficient sample size to calculate odds ratio APRDRG, All Patients Refined Diagnosis Related Groups

Variable	OR (95% CI)	p-Value
Age (years)		
2-4	Reference	
5-11	1.034 (0.654-1.636)	0.885
12-18	2.262 (1.470-3.480)	0.000
Gender		
Male	Reference	
Female	0.72 (0.62-0.83)	0.000
Median household income for patient's ZIP code		
0-25th percentile	Reference	
26-50th percentile	0.63 (0.53-0.75)	0.000
51-75th percentile	0.58 (0.48-0.71)	0.000
76-100th percentile	0.66 (0.51-0.85)	0.001
Type of insurance		
Medicaid	Reference	
Medicare	1.9 (0.44-8.24)	0.389
Private	0.52 (0.44-0.62)	0.000
Self-pay	0.68 (0.42-1.11)	0.126
No charge	0.00 (0.00-0.00)	0.999
Other	1.21 (0.87-1.69)	0.263
APRDRG severity of illness subclass		
No class specified	-^a^	
Minor loss of function (includes cases with no comorbidity or complications)	-^a^	
Moderate loss of function	Reference	
Major loss of function	1.305 (1.071-1.591)	0.997
Extreme loss of function	2.564 (1.560-4.215	0.997
Hospital urban-rural designation		
Large metropolitan areas with at least 1 million residents	Reference	
Small metropolitan areas with less than 1 million residents	0.93(0.80-1.09)	0.368
Micropolitan areas	0.94(0.66-1.35)	0.751
Not metropolitan or micropolitan (non-urban residual)	1.09 (0.62-1.92)	0.761
Teaching status of the hospital		
Metropolitan non-teaching	1.45 (1.161.80)	0.001
Metropolitan teaching	Reference	
Non-metropolitan	-^a^	
Comorbidities		
Thyroid disorder	0.77 (0.59-1.00)	0.048
Depression	1.74 (1.40-2.16)	0.001

Certain comorbidities with increased odds for DKA readmission were noted, such as thyroid disorder and depression. Other comorbidities considered in this study were obesity, hyperlipidemia, essential hypertension, non-essential hypertension, and substance abuse, but no statistical significance was found for them having increased odds for a 30-day DKA readmission.

## Discussion

As seen in the analysis of the 30-day DKA readmission data, several risk factors have been identified, as well as certain factors that influence the health outcome of a diabetic child.

Trends and risk factors 

In the population less than 20 years of age, diabetes is one of the most chronic diseases [17]. A surge has been noted in the number of people with T1DM and T2DM in this age group by 45% and 95%, respectively, from 2001 to 2017 [2]. The age of the child poses its own set of challenges when it comes to diabetes management. From infancy to preschool years, they are dependent on their caregivers for their diabetes care. The caregiver’s understanding of the disease and good communication between the provider and them plays an important role. Caregivers face immense stress and coping challenges with the child's condition. Making a schedule and strict monitoring of blood glucose levels multiple times in a day, the child's diet, and insulin dose calculation and administration is a daunting task. When a child enters into the toddler stage, food preferences kick in, and being able to work around it is challenging while trying to monitor content, timing, and amount of intake. This could lead to blood glucose level fluctuations due to irregularities in food intake. Once they start going to daycare or preschool, involving the staff there and educating them about the child’s condition is a challenge, and finding a place with staff trained to deal with this is even harder. Children at this age are not mature enough to voice their symptoms of hypoglycemia in the situation of miscalculated insulin doses or symptoms of hyperglycemia. Schools should have well-educated and trained staff as well to deal with a diabetic child. This burden is now shared with the child’s caregivers at home. As they get older and are involved in sports, their regimen has to be modulated accordingly. As the child moves into teenage years, their bodies are undergoing physical and mental changes. They get a sense of self-identity and crave more independence. They may tend to resent parent supervision, and a negotiation between the two to come up with an acceptable plan of diabetes management needs to be struck. Risk-taking behaviors tend to increase at this age. Alcohol and drug use cause problems in diabetes control and are risk factors for DKA [5,18,19]. These changes in the child’s life reflect in our analysis, with an increase in readmission trend in the early middle school age group and into the later teenage years of the study population.

Different factors seem to make the female gender an independent risk factor for poor glycemic control based on prior studies. Insulin sensitivity has shown to differ between males and females, with females having decreased sensitivity [20]. In one study, in the prepubertal and pubertal onset stages, females with T1DM were shown to have higher glycosylated hemoglobin A1C (HbA1C) levels. Cholesterol, low-density lipoprotein (LDL), and triglyceride levels were also found to be higher in girls, which can be attributed to the poorer glycemic control in them [21]. Eating disorder predisposition in females may be another contributing factor to fluctuating blood glucose levels leading to DKA. Eating disorders have been found to be more common in T1DM patients than the general population, with several studies showing females in different age groups being predisposed to them [22]. Our findings of observing more than half of our 30-day DKA readmissions being female children support prior literature.

Children from the lowest median household income category formed the greatest portion of 30-day DKA readmissions. Several factors can be contributory to this. Lower SES is related to lower educational level of the parents. This hinders their understanding of diabetes as a disease and its management. Family social support tends to be low. Stability of employment is low in this group. These factors put the children at risk of caregivers missing giving them insulin doses either due to not understanding the importance of timed doses or not having money to purchase the medication. They may not have means of transport to take the child for their checkups. They may also be living in areas in which a pediatric endocrinologist might not be available and the primary care physician may be the one managing the diabetes. They may not have the income to purchase healthier food options for the child. High-carbohydrate processed foods might be more freely available in the house, leading to poorer glycemic control in these children. Also, research has shown to link lower academic achievement and progress of children with their lower SES compared to their peers with a higher status. This affects their cognitive development, income, and health outcomes as they age [23].

In the fiscal year 2017, about 36.8 children were enrolled under Medicaid insurance across the nation [24]. Well-coordinated periodic care in clinics accordingly equipped has been shown in a prior study to reduce specialty and emergency room visits [25]. Federal-level policies, as seen in the past, that can help increase insured rates and increase preventative care are beneficial to those with chronic conditions especially when people with them are excluded from the coverage of insurance plans [26]. Expanding the insurance coverage to more of those in the FPL is key, and one study found that states with Medicaid expansion had a rise in more newly diagnosed diabetics compared to those states without expansion while another study found increased self-reported access to care and diabetes management in Medicaid expansion states [27,28]. In a study that looked into the trend of 30-day readmission of Medicaid versus privately insured patients from 2010 to 2017, the readmission rates tended to be higher in Medicaid-insured patients and there was a decline in the readmissions for both groups. Children with complex or chronic conditions formed the greater percentage of these readmissions [29]. In our study, the Medicaid-insured children were found to have higher odds and contributed to the majority of DKA 30-day readmissions, highlighting the importance of the need for insurance reforms to help improve healthcare disparities.

Urban areas tend to have more healthcare facilities equipped with pediatric floors and pediatric intensive care units than rural areas. Children from areas without these facilities are often transferred to such centers to get the medical management they need. This could have contributed to the higher readmissions observed at the urban hospitals in this study. It takes a multidisciplinary team to take adequate care of the child, and the more urban centers tend to have the needed additional staff of a pediatric endocrinologist, dietician, and social worker who can help with diabetes education and help in discharge planning for continued care and a plan for the child. Teaching hospitals usually have the primary and ancillary staff required to coordinate the education of the staff and the families better and lend the social support required to help procure the insulin, blood glucose level monitoring equipment, and medication delivery devices. They tend to have better access to an outpatient pediatric endocrinologist to help the child get continued postdischarge planned care for further monitoring and management, thus reducing DKA readmissions [30]. Depending on the location of the hospital and the frequency of DKA cases that they deal with, all hospitals may not be equipped equally to handle the medical care needed. This has been observed through our analysis that shows that non-metropolitan or micropolitan hospitals and metropolitan non-teaching hospitals have higher odds of having 30-day DKA readmissions.

In one study that looked into the years 2009-2010, the unplanned 30-day unadjusted readmission rate for all hospitalized children was 6.5% [31]. Another study that specifically looked at 30-day unplanned readmissions for DKA in children from 2010 to 2014 found a readmission rate of 4.6%. Our study found a readmission rate of 4.3% in 2017, which has not changed dramatically from that of prior years, though it is lower than the all-cause readmission rate. The mean LOS in that study for those with readmission versus those without was 2.77 and 2.45 days, respectively [32]. In our study, the mean LOS of those with readmission versus no readmission was 2.06 versus 2.28 days. Those who did not need readmission also had higher hospital costs associated with the inpatient stay. Those with a longer LOS may be receiving enough time for an efficient diabetic education in order to have better planned care that could prevent another readmission.

In one retrospective study, it was noted that children with DKA or severe diabetes having chronic psychiatric illnesses ended up with longer hospital stays and more hospital charges [33]. A study that looked into readmissions noted that in patients younger than 35 years, the odds for a readmission were increased in those with depression and substance or alcohol abuse [34]. Another study that looked into the pediatric age group of 2-18 years also noted the 30-day readmission rate to have higher odds in those with depression [18]. Our study found higher odds as well of a 30-day DKA readmission in those with depression, thus observing a trend that has not changed over the years. Another finding that has been noted in the literature is that in youth with diabetes aged 10-21 years, higher HbA1C levels and frequent emergency room visits were associated with a depressed mood. Physician screening for depressed mood in diabetic children in the clinic especially in those with poor glycemic control and appropriate counselling and management places an important role in their health outcomes [35]. In our study, one interesting observation was increased odds of a 30-day DKA readmission in those with thyroid disorder. In one study, it was noted that there may be an interplay of factors for diabetes mellitus and thyroid disorder that put these children more at risk. A higher degree of acidosis was associated with children who had lower levels of free thyroid hormones and euthyroid sick syndrome [36]. In one pediatric study, the prevalence of hypothyroidism was higher in children and adolescents with T1DM [37].

Preventative measures

Continued and coordinated postdischarge care is of utmost importance in diabetic patients to prevent readmissions and improve long-term health outcomes. In today's day and age of electronic communications, there have been studies that have demonstrated decreased healthcare utilization and costs by sending text messages to the patient’s caregivers on a frequent basis that addressed diabetes education, targeted blood glucose level monitoring, and insulin usage monitoring [38,39]. Adherence to self-care is a commonly found barrier in teenage patients, and one study showed good outcomes in maintaining glycemic control in those who participated in a web-based diabetes messaging system [40]. Group visits have been found to be beneficial in a study that included T2DM patients. It is a holistic approach by a team of medical professionals that assesses and addresses the educational needs, sets specific goals, evaluates the patient’s learning curve, and assesses the outcome and efficacy of the whole intervention [41]. Telemedicine has proven to be an important tool in patient care especially with the coronavirus disease 2019 (COVID-19) pandemic. Diabetes education and monitoring with the help of telemedicine will make diabetes care more accessible even in areas in which a physician may not be physically available, thus cutting travel time and costs and making it more convenient for the patient and their families [42]. Trying to set up after-hour availability for the patients and their families will get them better healthcare access. Acute infectious diseases are common in children and can disrupt their glycemic control. Efficiently managing them through their days of acute illness is key in preventing a readmission.

Depending on the etiology of the illness, the child may have associated hypo- or hyperglycemia with it. Family should have an established way of communication with the healthcare provider. More frequent blood glucose monitoring and checking urine for ketones help remotely manage the child. Urine strips for home urine ketone detection are an economical investment for the family. Insulin dosage modulation according to the acute illness is key. Most often, the patient is not given or does not take the insulin in such instances. Maintaining hydration of the patient is key, and in cases of illnesses that may cause hypoglycemia, having glucose tablets on hand or sweets and hydrating electrolyte solutions is vital. Educating a diabetic patient and their family about sick day supplies helps them be more prepared in times like these. The principles for management of sick diabetics are the same for those on insulin injection versus those on insulin pumps. In illnesses associated with hyperglycemia risk, since the diabetics on an insulin pump use rapid or short-acting insulin, through it they are more prone to DKA if the acute illness is not addressed in a timely manner [43]. For patients on an insulin pump, providing them with a pump failure backup insulin plan is vital. In one study, reduction in the prescription co-pay for diabetic medications showed an increased adherence to and use of these medications [44]. In patients who appear to be non-compliant with their insulin regimen or have not been taking it, it is of utmost importance to group with the family and find the reason such as economical barrier or other reasons that could be modifiable. Referring them to community services as needed will help better outcomes.

In patients with mental health disorders, making sure they have an established go-to healthcare provider is essential. Chronic health conditions are a stress to the patient and their families, and diabetics with thyroid disorder and depression have been shown to have higher odds of a 30-day unplanned DKA readmission. In one study, it was noted that those who have recurrent DKA have associated increased levels of anxiety and stress and find emotion regulation hard [45]. Medicare has brought about a value-based purchasing program, the Hospital Readmissions Reduction Program (HRRP), which helps reduce avoidable readmission by encouraging better communication and coordination of patient care by hospital staff that results in improved discharge plans. This initiative currently includes six medical conditions, and DKA is not one of them. This program links the quality of patient care to payments [46].

Limitations

NRD does not provide data on the patient’s race and or ethnicity or geographical distribution, which hinders studying certain sociodemographic factors. HbA1C levels of the patient are not available in the database to assess the glycemic control trends of a given patient or of all the patients that met the inclusion criteria. Patient data are not linked over consecutive years when a longer study period is chosen. NRD does not permit the determination of regional variations within the dataset or in out-of-state admissions. Children admitted as an outpatient or inpatient observation status are not captured by the NRD, and the 30-day readmissions may be higher than those observed in our study. Unmeasured confounders may exist, and the results of our study as with any observational data cannot suggest causal relationships. Postdischarge follow-up data of the patients are not available. Postdischarge care plays a crucial role as an influencing factor for a readmission.

## Conclusions

Despite the steady rise in T1DM and T2DM among the younger population of the USA from 2001 to 2017, there has not been much of a change in the trend and risk factors contributing to the 30-day unplanned DKA readmissions over the years. The LOS for those who did not get readmitted within 30 days was longer than those who did. This could reflect more comprehensive care and discharge planning that may have prevented them from readmit. Comorbidities such as depression and thyroid disorder had increased odds of a 30-day readmission, and counselling for mental health wellness and coordinated medical care continues to play a critical role. Insurance coverage reforms will be needed to make medication and resources more accessible to the patients and their families. Innovative ways to increase awareness and compliance using web-based technologies can be explored further especially for the young adolescents in the day of smartphone usage, who were observed to have higher odds of 30-day DKA readmissions among the age groups with a sharp rise of cases in them. Utilizing these data, quality improvement studies can be carried out in the inpatient care units to help reduce readmission rates. Diabetes mellitus is a chronic disease that demands a team effort from the patient, family, healthcare personnel, insurance companies, and lawmakers. There is scope for a lot of improvement with the way our patients are being managed, and a more holistic approach needs to be devised.
